# Active crocodiles are less sociable

**DOI:** 10.1098/rstb.2022.0528

**Published:** 2024-09-04

**Authors:** Cameron J. Baker, Barbara Class, Ross G. Dwyer, Craig E. Franklin, Hamish A. Campbell, Terri R. Irwin, Céline H. Frère

**Affiliations:** ^1^ Research Institute for Environment and Livelihoods, Charles Darwin University, Darwin, Northern Territory 0815, Australia; ^2^ The School of the Environment, The University of Queensland, Brisbane, Queensland 4072, Australia; ^3^ Ludwig-Maximilians-Universität München, Munich 80539, Germany; ^4^ School of Science, Technology and Engineering, University of the Sunshine Coast, Sippy Downs, Queensland 4556, Australia; ^5^ Australia Zoo, Steve Irwin Way, Beerwah, Queensland 4519, Australia

**Keywords:** acoustic telemetry, *Crocodylus porosus*, behavioural syndrome, animal personality, double hierarchical model, sociability

## Abstract

How animals move and associate with conspecifics is rarely random, with a population’s spatial structure forming the foundation on which the social behaviours of individuals form. Studies examining the spatial–social interface typically measure averaged behavioural differences between individuals; however, this neglects the inherent variation present within individuals and how it may impact the spatial–social interface. Here, we investigated differences in among-individual (co)variance in sociability, activity and site fidelity in a population of wild estuarine crocodiles, *Crocodylus porosus,* across a 10-year period. By monitoring 118 crocodiles using coded acoustic transmitters and an array of fixed underwater receivers, we discovered that not only did individual crocodiles repeatably differ (among-individual variation) in each behaviour measured but also in how consistently they expressed these behaviours through time (within-individual variation). As expected, crocodile activity and sociability formed a behavioural syndrome, with more active individuals being less sociable. Interestingly, we also found that individuals that were either more sociable or displayed greater site fidelity were also more specialized (lower within-individual variation) in these behaviours. Together, our results provide important empirical evidence for the interplay between spatial, temporal and social individual-level behavioural variation and how these contribute to forming behavioural niches.

This article is part of the theme issue ‘The spatial–social interface: a theoretical and empirical integration’.

## 1. Introduction

How animals move throughout their environment is rarely random [1], with these non-random patterns in space forming the population’s underlying spatial structure [2]. To reduce the costs of social conflict, the associations between conspecifics are also typically non-random, with individuals displaying distinct preferences or aversions towards conspecifics within their environment [3–6]. As shared space use is the prerequisite for associations to occur [7], the spatial structure of a population forms the foundation on which the social behaviours and complexity (i.e. social organization, social structure, mating and care system) of a species develop and evolve [8]. Indeed, many studies have demonstrated this inherent correlation between the spatial and social behaviours of animals [9]. For instance, how individuals move throughout their environment and access resources can be influenced by the presence or absence of conspecifics [10,11]. Furthermore, individuals which are more active and occupy larger home ranges may encounter more social partners, while by contrast, the associations of territorial species are constrained by the size and location of individual territories [12,13]. Understanding this interplay between the spatial and social phenotypes of individuals has important ecological and evolutionary implications, including pathogen transmission [14], population dynamics [10,15,16] and ultimately the evolution of social systems.

Repeatable between-individual differences in behaviour, otherwise known as animal personality, are ubiquitous across the animal kingdom [17]. These between-individual differences have been observed for a wide range of behaviours, including activity [18,19], home range size and site fidelity [20], sociability [21,22] and aggression towards conspecifics [23]. Individuals may also vary in how consistently (i.e. the degree of within-individual variation) they adopt specific behavioural phenotypes over time in response to shifts in intrinsic (i.e. maturity status and physiological condition) or extrinsic (i.e. time of year and conspecific density) factors [24–26]. Individuals that show low within-individual variation in a given phenotype may be specialists in that behavioural trait, while those that show higher within-individual variation are more generalist.

How specialized individuals are within a specific behaviour often correlates to their average expression of that behaviour (i.e. personality) [25,26]. For instance, in foraging great cormorants (*Phalacrocorax carbo*), individuals who dive for shorter periods consistently apply this foraging tactic. By contrast, those individuals with longer dive durations are more flexible in their foraging behaviour [25]. An individual’s degree of behavioural specialization may also, in turn, influence their expression of other behaviours through the formation of behavioural syndromes (i.e. correlations between functionally distinct behaviours) [27,28]. For example, in red squirrels (*Tamiasciurus hudsonicus*), individuals specializing in high site fidelity are often more sociable, owing to a greater familiarity and willingness to associate with conspecifics [29,30]. Thus, it is crucial to understand not only how individuals within a population differ in the behaviours they adopt but also in how specialized they are within these phenotypes. However, the majority of studies examining how the spatial and social phenotypes of individuals are correlated have only focused on quantifying the average behavioural differences between individuals. In doing so, we are ignoring the subtle ways in which individuals differ in their spatial and social behaviours through time and, in turn, how this may be influencing the interface between the spatial and social behaviours of individuals.

Previous studies have demonstrated that estuarine crocodiles (*Crocodylus porosus*) are not homogenous in their space use [31–33]. While considerable variation was present within the same population, individuals generally adopted either a nomadic movement tactic characterized by high daily activity and low site fidelity, or a resident movement tactic characterized by low daily activity and high site fidelity [34]. Estuarine crocodiles are also considered to be the least social and most aggressive crocodylian [35], with associations and conflict between conspecifics potentially leading to severe injury (i.e. limb or tail loss and lacerations) or death [34,36]. Despite this apparent social intolerance, estuarine crocodiles have been found to not only display non-random spatial structuring, with individuals actively maintaining spatial overlaps with conspecifics [37], but also the formation of social communities and stable associations between conspecifics [38]. An individual’s movement tactic has also been observed to influence the stability of their social environment. Crocodiles adopting a resident movement tactic display highly consistent spatial overlaps with conspecifics. By contrast, those undertaking a nomadic movement tactic displayed far more dynamic spatial overlaps with conspecifics [37]. While it has been suggested that these two movement tactics represent alternative behavioural phenotypes that crocodiles adopt to navigate their social environments, no studies, to our knowledge, have examined how the spatial (i.e. activity and site fidelity) and social (i.e. sociability) behaviours of crocodiles correlate at the individual scale.

Here, we quantified the degree of among- (behavioural phenotypes) and within-individual (behavioural consistency) variation in sociability, daily activity and site fidelity for a wild population of estuarine crocodiles. We investigated whether an individual’s behavioural phenotype correlates with their consistency across consecutive tracking months and whether behaviours were correlated at the among-individual level, thus forming behavioural syndromes. We predicted that crocodiles would display both among- and within-individual variation in spatial and social phenotypes. Specifically, we hypothesized that behavioural phenotypes are correlated with behavioural consistency, with more sociable and site-attached individuals being more consistent through time, while more active individuals would be less consistent. Finally, we predicted that an individual’s degree of sociability would be positively correlated with both their daily activity and the degree of site fidelity.

## 2. Methods

### (a) Crocodile capture and tagging

We used movement data collected from estuarine crocodiles (*C. porosus, n =* 118) captured from the Wenlock River, Cape York, Queensland, Australia (latitude, longitude: −12.385, 142.179). Estuarine crocodiles were captured following the methods previously detailed in [37]. Once captured, crocodile sex and total body length (TL, m) were recorded. Individuals were then surgically implanted with a coded acoustic transmitter (V16T or V13T, https://www.innovasea.com) behind the left forelimb following surgical procedures outlined in Franklin *et al.* [39]. A projected battery life of 7–10 years and small size (diameter: 10 mm V13T, 16 mm V16T) permitted the recording of movements across a wide size range of individuals (0.86–4.64 m TL) across multiple years (max tag life = 10 years). An array of hydrophone receivers (VR2-W, https://www.innovasea.com) was placed throughout a 100 km section of the Wenlock River to monitor the movements of tagged crocodiles (electronic supplementary material, figure S1). Receivers were placed approximately 2–5 km apart and 2–20 m from the riverbank and approximately 1 m below the water surface. As the pulse transmission rate of transmitters was set to transit randomly between 90 and 120 s, and the detection radius of each receiver was 400 m with river width rarely exceeding 100 m wide, it would be unlikely for crocodiles to pass by a receiver without being detected.

### (b) Determining crocodile behaviours

To estimate sociability, we first determined an individual’s social environment. As the social environment is composed of all the conspecifics an individual may interact with (or avoid) [5,37,40,41], we quantified an individual’s social environment as the number of tagged conspecifics detected at any receivers that the focal individual was detected at within each calendar month (i.e. January 2019 and February 2019). Crocodiles were considered to be associating with tagged conspecifics if they were observed simultaneously co-occurring at hydrophone receivers within a 4 min sampling window following Baker *et al.* [38]. Crocodile sociability was then calculated as the proportion of conspecifics an individual was observed associating with (observed associates) over the number of individuals present within their social environment (potential associates) per calendar month.

To obtain a metric of activity, we calculated the mean river distance travelled by a crocodile per day. For this, raw tag detections were converted into short-term centres of activity (COA) using the *VTrack* [42] package in R [43]. COAs account for potential biases in movement estimates when using an array of fixed hydrophone receivers by determining the weighted average position of an individual within a user-defined temporal bin [44]. As the movements of crocodiles are restricted to the boundaries of the river system, COA estimates were assigned to the corresponding closest point on the river using the *sf* [45] R package. We then calculated the distance between each successive COA following the methods detailed in Dwyer *et al.* [33], with the distances then summed by day and averaged by month.

To estimate site fidelity, we used least cost utilization distributions (lcUD) to generate monthly home range estimates (95% lcUD) for individuals following the methods described in [37,46]. A minimum of five unique locations was required per month to generate home range estimates. We then estimated the overlap of our consecutive monthly home range estimates using the volume of intersection method. Doing so provided a simple measure of within-individual home range overlap ranging from 0 (when two home ranges have no overlap) to 1 (when two home ranges are identical). We only included crocodiles for which measures of all three behaviours (sociability, activity and site fidelity) were recorded.

### (c) Model fitting

Following Hertel *et al.* [26], we fitted multivariate double-hierarchical generalized linear models (DHGLMs) with sociability, activity and site fidelity as the response variables using the brms [47] R package based on the Bayesian software Stan [48]. DHGLMs are composed of both a ‘mean model’ and a ‘dispersion model’ component, which can fit both fixed and random effects. DHGLMs are therefore able to simultaneously estimate whether individuals differ in their mean expression of a behaviour (i.e. behavioural phenotype and mean component) along with whether they differ in their residual intra-individual variation (rIIV) around their behavioural mean (i.e. behavioural consistency and dispersion component). Importantly, the dispersion model is estimated on the log scale, ensuring that the population mean residual variation (the intercept of the dispersion model) and the individual rIIV are always positive [49]. We further estimated the correlation between the random intercepts of the mean and dispersion models (i.e. correlation between the behavioural mean and rIIV) and the among-individual correlation of behavioural means (i.e. behavioural syndrome) for each response variable. As inferences about residual variation can be misleading if data are skewed prior to modelling [50], the response variable sociability and activity were cube root transformed before the analysis to normalize their distributions. All response variables were then analysed using a Gaussian distribution after being standardized (mean = 0, s.d. = 1). As behavioural measures for either activity or site fidelity were missing for some months, following Hertel *et al.* [26], we fitted each model to the subset, maximizing the number of months with behavioural measures (sociability = 2826, activity = 2824, site fidelity = 1454). Among-individual differences and regression coefficients were estimated on the subset of months for which data of each behavioural measure were available, while among-individual correlations in behavioural means and rIIV were based on the months that measures of sociability, activity and site fidelity were all available. Separate models were created to determine whether sex-based differences in individual behavioural means and rIIV (i.e. behavioural consistency) were present within the population. As no differences were present between the sexes, only the full model results are reported in the text. Results of the male and female models are available in the electronic supplementary material, tables S1 and S2.

#### (i) Mean model

We included sociability, activity and site fidelity as response variables with identical fixed and random effects model structures into a multivariate mixed model to model for differences in average behaviour. For all response variables, we added a population intercept (*β*_0_) and controlled for the effects of month (as a factor, e.g. May and June), crocodile total length (as a linear effect, *Z*-transformed to mean = 0 and s.d. = 1) and number of detections per month (as a linear effect, *Z*-transformed to mean = 0 and s.d. = 1) by fitting these terms as fixed effects. As we found no differences in the individual behavioural means and consistency (rIIV) between the sexes in any of the behaviours examined, we excluded sex as a fixed effect to avoid overfitting the models. To avoid violating the assumption of normality within the residuals, the number of detections per month was log-transformed. We fitted crocodile identity (ID) and study year as random effects to estimate among-individual and among-year variation in the mean responses.

#### (ii) Dispersion model

We modelled the residual standard deviation of each sociability, activity and site fidelity measure (on the log scale). We structured the dispersion model to mirror the mean model by adding a population intercept (*ϒ*_0_) and included month, crocodile total length and number of detections per month as fixed effects. We fitted random intercepts for crocodile ID and study year to estimate the among-individual and among-year variation in residuals. To assess the consistency of individual differences in predictability through time, we used the hyperparameter *ω* ^2^ as it reflects on the log scale how strongly individuals differ in their rIIV [49,51]. We estimated the full correlation matrix among all random effects, i.e. the random intercepts of the multivariate mean model and the random intercepts of the multivariate dispersion model on both the study year (year) and individual (crocodile ID) level following the equations provided in [26]. Given the aims of this research, we focused on the correlations between behavioural phenotype and consistency, along with the cross-trait among-individual correlation of behavioural means (i.e. behavioural syndrome) in the main text. We report the full correlation matrix of the fitted model in the electronic supplementary material, table S3.

#### (iii) Model validation

Models were run using uninformative priors. We ran four chains to evaluate convergence which were run for 8000 iterations, with a warm-up period of 6000 iterations and a thinning interval of 2. Therefore, all estimated model coefficients and credible intervals were based on 2000 posterior samples and had satisfactory convergence diagnostics with *$\hat{R}$* < 1.01 and effective sample sizes greater than 1000 [52]. Posterior predictive checks recreated the underlying Gaussian distributions for the degree of sociability, daily activity and degree of site fidelity. Model performance was further validated using a sensitivity analysis which varied the minimum required observations for inclusion in the study. Model coefficients and credible intervals remained stable throughout the analysis, indicating a negligible influence of an individual’s number of observations on model performance (electronic supplementary material, table S4). We report the mean and 95% credible intervals, calculated as the highest posterior density intervals, for all parameters in our statistical models to assess whether parameters were statistically different from 0. All analyses were completed using R 4.1.0.

### (d) Metrics of interest

#### (i) Behavioural phenotypes and repeatability

Repeatability represents a standardized metric of the total variation of a trait that is attributable to between-individual differences, allowing comparisons between traits and sexes [53]. We calculated repeatability following the equation:


$$R=V_{crocodileID}/(V_{crocodileID}+V_{year}+V_{residual}),$$

where *V*_crocodile ID_ is the between-individual variation, *V*_year_ is the between-year variation and *V*_residual_ is the residual variation. Here, unlike in classic mixed effects models, the residual variation (*V*_residual_) refers to the population intercept of the residual model (*ϒ*_0_). As the dispersion model uses a log scale to estimate rIIV, we converted this estimate into a variance by taking its exponent and then squaring the resulting value. We extracted the behavioural phenotypes for sociability, activity and site fidelity as the mean and 95% credible interval of the posterior distribution of the random effect for each individual. As the model was fitted using *Z*-transformed response variables, to facilitate biological interpretation, we added the population-level intercept to the random intercepts and then back-transformed the resulting ‘realized’ behavioural phenotypes onto their original scale.

#### (ii) Individual consistency

We estimated the individual variation in consistency as the coefficient of predictability (CV_*p*_) following the equation:


(2.1) $${CV}_{p}=\sqrt{\left(exp\left(w^{2}\right)-1\right)}.$$

CV_*p*_ is a standardized metric quantifying the degree of variation in within-individual predictabilities, allowing us to compare and contrast differences in within-individual variation across behaviours and between sexes [49]. We then extracted the posterior distribution of the rIIV for each level of the random intercepts of crocodile ID as an indicator of individual consistency. When rIIV is high, the residual variation around an individual’s behavioural mean is high indicating behavioural variability (inconsistency). As rIIV is estimated on the log scale, to facilitate biological interpretation, we added the population-level mean standard deviation (intercept) and then exponentiated and back-transformed the resulting ‘realized’ rIIV.

#### (iii) Correlation between behavioural phenotypes and consistency

We extracted the mean and 95% credible intervals for the among-individual correlation of behavioural means and predicted standard deviation of residual variance of male and female crocodiles for each of the behaviours examined.

#### (iv) Behavioural syndromes

We extracted the mean and 95% credible intervals for the among-individual correlation of behavioural means of sociability, daily activity and site fidelity.

### (e) Home range size and associates

An individual’s number of social partners typically increases as they become more active and occupy larger home ranges [12,13]. To determine whether the behavioural syndrome between active and sociability is not a by-product of our methodology for calculating sociability, we investigated the influence of home range size on the number of conspecific individuals encountered per month. For this, we determined the number of potential (conspecifics within their social environment) and observed (conspecific individuals were observed associating with) associate individuals encountered per month following the methods above. Monthly home range size (km^2^) was extracted from the monthly 95% lcUD home range estimates determined above. We then fitted a generalized linear mixed model with a Poisson distribution using the brms R package, with the number of associates as the response variable. The interaction between home range size and association type (potential and observed) was included as predictor variables, and crocodile ID and study year were included as random effects.

## 3. Results

We analysed data from 118 estuarine crocodiles (0.84–4.67 m total length; males = 78, females = 40) monitored over a 10-year period within the Wenlock River. Individuals were monitored for 3.7 years on average (± 2.8 years s.d.; electronic supplementary material, table S5). For a given individual, we determined their degree of sociability for 2–115 months (25 ± 21 months; mean ± s.d.), daily activity for 2–115 months (25 ± 21 months) and the degree of site fidelity for 1–98 months (13 ± 15 months). On average, crocodiles associated with 7.08% of their social environment (back-transformed *β*_0_ intercept, table 1). A distinct within-year cyclic trend was also present, with the degree of sociability peaking during September (table 1).

**Table 1.** Estimates and 95% credible intervals (in brackets) of fixed and random effects on individual sociability, activity (km d^−1^) and site fidelity (mean model) and residual standard deviation of sociability, activity and site fidelity (dispersion model) of estuarine crocodiles (*C. porosus*). (Variation in and correlation between mean behaviour and residual standard deviation of behaviour (rIIV) were estimated among individuals. Estimates are based on double hierarchical mixed models. Bold indicates estimates for which 95% credible intervals did not include zero.)

	sociability	activity (km d^−1^)	site fidelity
**mean model**			
*fixed effects*			
intercept	**−0.20 (−0.31 to −0.09)**	**0.33 (0.17 to 0.49)**	−0.00 (−0.17 to 0.17)
month			
February	0.01 (−0.18 to 0.20)	−0.07 (−0.22 to 0.09)	−0.14 (−0.40 to 0.12)
March	**−0.37 (−0.56 to −0.18)**	−0.08 (−0.25 to 0.09)	−0.28 (−0.60 to 0.04)
April	0.12 (−0.05 to 0.28)	0.04 (−0.09 to 0.18)	**−0.52 (−0.77 to −0.27)**
May	0.02 (−0.15 to 0.11)	0.07 (−0.06 to 0.20)	**−0.36 (−0.57 to −0.14)**
June	0.09 (−0.03 to 0.22)	−0.12 (−0.25 to 0.01)	**−0.39 (−0.60 to −0.18)**
July	**0.20 (0.08 to 0.32)**	**−0.22 (−0.34 to −0.09)**	−0.18 (−0.39 to 0.03)
August	**0.29 (0.18 to 0.41)**	**−0.13 (−0.24 to −0.01)**	**−0.41 (−0.61 to −0.21)**
September	**0.39 (0.28 to 0.49)**	−0.06 (−0.18 to 0.05)	−0.14 (−0.32 to 0.04)
October	**0.38 (0.27 to 0.48)**	−0.02 (−0.13 to 0.10)	0.02 (−0.15 to 0.19)
November	**0.27 (0.16 to 0.38)**	−0.07 (−0.18 to 0.06)	−0.02 (−0.20 to 0.15)
December	**0.24 (0.13 to 0.35)**	0.00 (−0.12 to 0.11)	−0.06 (−0.23 to 0.12)
total length (m)	0.02 (−0.04 to 0.07)	**0.34 (0.22 to 0.46)**	**0.13 (0.01 to 0.24)**
number of detections	**0.64 (0.61 to 0.68)**	**0.15 (0.11 to 0.19)**	**0.11 (0.03 to 0.20)**
*random effects*			
crocodile ID			
s.d._intercept.crocodileID_	**0.27 (0.22 to 0.32)**	**0.58 (0.50 to 0.67)**	**0.49 (0.41 to 0.59)**
year			
s.d._intercept.year_	**0.04 (0.01 to 0.09)**	**0.11 (0.06 to 0.20)**	0.04 (0.00 to 0.11)
**dispersion model**			
*fixed effects*			
intercept	**−0.38 (−0.51 to −0.26)**	**−0.28 (−0.41 to −0.15)**	**−0.25 (−0.40 to −0.08)**
month			
February	**0.21 (0.05 to 0.37)**	0.02 (−0.14 to 0.19)	0.15 (−0.10 to 0.41)
March	0.11 (−0.05 to 0.28)	0.01 (−0.16 to 0.18)	0.20 (−0.11 to 0.53)
April	**0.16 (0.01 to 0.32)**	−0.04 (−0.21 to 0.11)	**0.35 (0.12 to 0.59)**
May	−0.01 (−0.16 to 0.13)	0.01 (−0.14 to 0.15)	**0.23 (0.03 to 0.43)**
June	0.02 (−0.12 to 0.17)	0.02 (−0.13 to 0.17)	0.20 (0.00 to 0.40)
July	−0.05 (−0.19 to 0.10)	−0.04 (−0.18 to 0.11)	**0.21 (0.02 to 0.42)**
August	−0.07 (−0.22 to 0.06)	−0.13 (−0.27 to 0.01)	0.09 (−0.11 to 0.28)
September	**−0.37 (−0.51 to** −**0.23)**	**−0.15 (−0.29 to −0.01)**	−0.00 (−0.19 to 0.18)
October	**−0.40 (−0.53 to −0.27)**	**−0.18 (−0.35 to −0.04)**	−0.04 (-0.22 to 0.14)
November	**−0.21 (−0.35 to −0.08)**	−0.14 (−0.28 to 0.00)	−0.08 (−0.27 to 0.10)
December	**−0.28 (−0.42 to −0.14)**	**−0.12 (−0.26 to 0.01)**	−0.02 (−0.22 to 0.17)
total length (m)	−0.03 (−0.10 to 0.04)	−0.05 (−0.13 to 0.02)	**−0.08 (−0.16 to −0.01)**
number of detections	**−0.30 (−0.34 to −0.25)**	**−0.22 (−0.26 to −0.17)**	−0.04 (−0.11 to 0.03)
*random effects*			
crocodile ID			
*ω* ^2^_crocodileID_	**0.29 (0.24 to 0.36)**	**0.31 (0.25 to 0.37)**	**0.26 (0.20 to 0.34)**
*r*_intercept.crocodileID − *ω*crocodileID_	**−0.56 (−0.73 to −0.35)**	0.01 (−0.22 to 0.23)	**−0.66 (−0.82 to −0.46)**
year			
*ω* ^2^_intercept.year_	**0.08 (0.02 to 0.15)**	**0.08 (0.02 to 0.16)**	0.07 (0.00 to 0.17)

At the population level, crocodiles moved on average 3.17 km d^−1^ (back-transformed *β*_0_ intercept, table 1). Time of year was not associated with the daily activity of crocodiles (table 1). Body size was positively correlated with an individual’s activity, with larger individuals being more active than smaller individuals (table 1). Finally, on average, crocodiles retained 59% of their home range between consecutive months (back-transformed *β*_0_ intercept, table 1). Body size also positively correlated with an individual’s degree of site fidelity, with larger individuals displaying a greater degree of site fidelity than smaller individuals (table 1). Site fidelity was found to decrease between April and June before then remaining stable throughout the remainder of the year (table 1).

### (a) Behavioural phenotypes and repeatability

**Figure 1.**
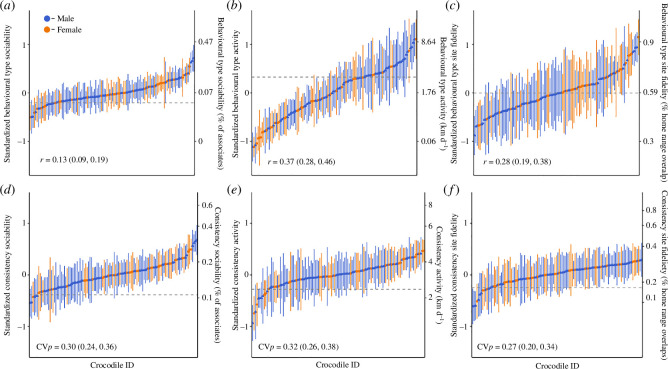
Behavioural phenotypes (*a–c*) and behavioural consistency (*d–f*) for the degree of sociability (*a,d*), daily activity (*b,e*) and degree of site fidelity (*c,f*) in 118 estuarine crocodiles (*C. porosus*) monitored over 10 years. Circles and error bars indicating individual estimates and 95% credible intervals, respectively. Dashed line represents the population average. Behaviours were *Z*-transformed, and rIIVs are estimated on a log scale; the second *y*-axis shows estimates in original units (i.e. proportion of conspecifics, km d^−1^, % of home range overlaps).

We found significant between-individual variation in crocodiles for all three behaviours (table 1). Between 13 and 37% of the observed variation across, the three behaviours could be explained by between-individual differences (figure 1). Daily activity was found to be the most repeatable of the three behaviours (*r* = 0.37 (0.28 to 0.46); figure 1). By contrast, an individual’s degree of sociability was found to be the least repeatable of the behaviours (*r* = 0.13 (0.09 to 0.19); figure 1).

### (b) Behavioural consistency

The population-level mean rIIV was estimated to be 16% for sociability, 3.49 km d^−1^ for activity and 23% for site fidelity (back-transformed intercepts of dispersion models exp(*y_0_*), table 1). Crocodiles became more consistent in their degree of sociability between September and December (table 1). By contrast, crocodiles became less consistent in their degree of site fidelity between April and July (table 1). Body size was found to only influence individuals’ degree of site fidelity, with larger crocodiles being more consistent in their degree of site fidelity than smaller individuals (table 1). The predicted standard deviation from the mean rIIV for sociability (*ω* ^2^_crocodileID_ table 1; CV_*p*_ = 0.30 (0.24 to 0.36)), daily activity (*ω* ^2^_crocodileID_ table 1; CV_*p*_ = 0.32 (0.26 to 0.38)) and site fidelity (*ω* ^2^_crocodileID_ table 1; CV_*p*_ = 0.27 (0.20 to 0.34)) varied across individual crocodiles, demonstrating differences in the behavioural consistency of each behaviour across individuals (figure 1).

### (c) Behavioural phenotype correlates with behavioural consistency

**Figure 2.**
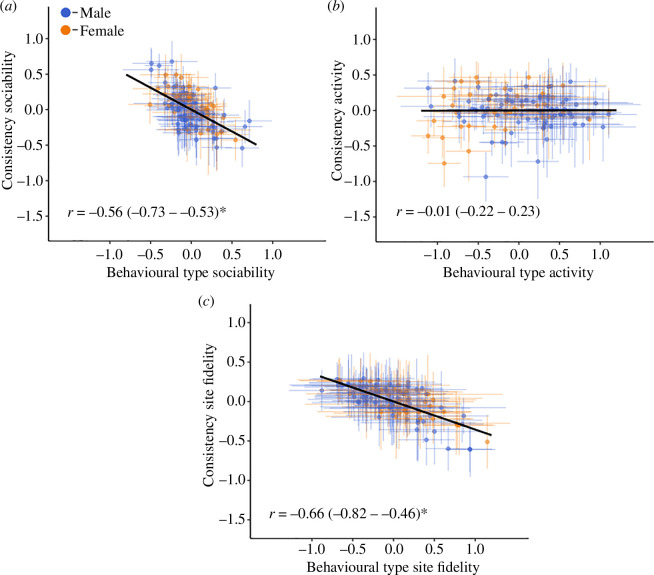
Among-individual correlation (*r*) between behavioural phenotype and behavioural consistency in the degree of sociability (*a*), daily activity (*b*) and degree of site fidelity (*c*). Circles and error bars indicating individual estimates and 95% credible intervals, respectively. Asterisks (*) represent significant results.

An individual’s behavioural phenotype and their consistency was negatively correlated for their degree of sociability (*r* = −0.56 (−0.73 to −0.35); table 1; figure 2*a*). Individuals which displayed a greater degree of sociability were more consistent in their behaviour, as indicated by a lower rIIV value. Similarly, crocodiles which displayed a greater degree of site attachment were also more consistent in this behavioural phenotype across months, while those individuals that were less site attached were less consistent in this phenotype (*r* = −0.66 (−0.82 to −0.46); table 1; figure 2*c*). Overall, no correlation was observed for an individual’s degree of activity between their behavioural phenotype and their consistency (*r* = 0.01 (−0.22 to 0.23); table 1; figure 2*b*).

### (d) Behavioural syndromes

**Figure 3.**
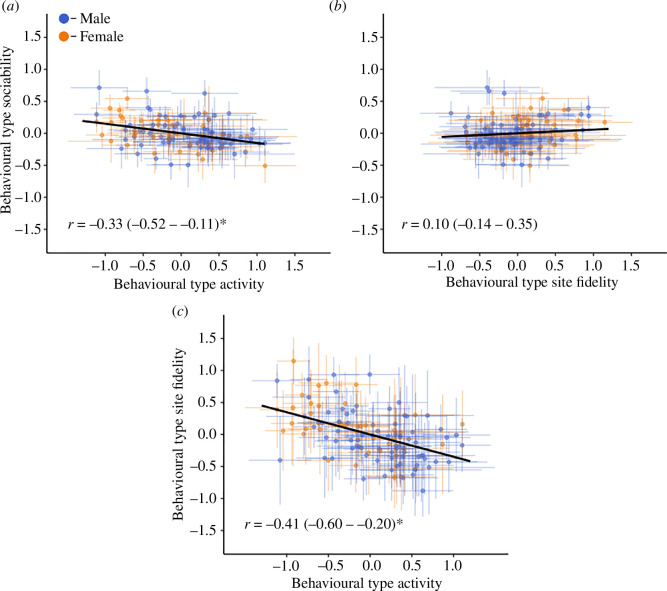
Among-individual correlations (*r*) between the (*a*) sociability, (*b*) daily activity and (*c*) site fidelity behavioural phenotypes (i.e. behavioural syndromes). Circles and error bars indicating individual estimates and 95% credible intervals, respectively. Asterisks (*) represent significant results.

Daily activity was found to negatively correlate with both an individual’s degree of sociability (*r* = −0.33 (−0.52 to −0.11)) and site fidelity (*r* = −0.41 (−0.60 to −0.20)). Crocodiles which travelled greater distances per day displayed both lower degrees of sociability and site fidelity (figure 3*a,c*). No correlation was present between an individual’s degree of sociability and site fidelity (*r* = 0.10 (−0.14 to 0.35); figure 3*b*).

### (e) Home range size and associates

**Figure 4.**
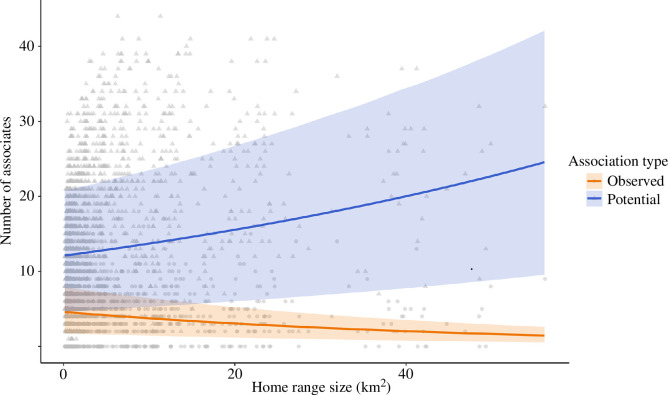
The correlation between individual home range size (km^2^) and the number of associates that estuarine crocodiles (*C. porosus*) encounter per month. Blue represents the potential associates (conspecifics within their social environment) and red represents the number of conspecific crocodiles that were observed associating with. Lines represent mean model estimates, and the ribbon represents the 95% credible intervals.

We found a significant interaction between home range size and association type (potential and actual) when predicting the number of conspecifics that individuals encountered per month (0.23 (0.12 to 0.25)). While the number of potential associates (overlapping conspecifics) increased with an individual’s home range size, we found that tagged crocodiles with larger monthly home ranges instead had fewer observed associations with conspecifics per month (figure 4).

## 4. Discussion

By monitoring the movement behaviour and co-occurrences of 118 estuarine crocodiles for 10 years, we investigated the degree of both among-individual (i.e. behavioural phenotypes/personalities) and within-individual (i.e. behavioural consistency) behavioural variation in this wild population. We found that consistent with previous work on other animal species, estuarine crocodiles display repeatable individual differences in their spatial and social phenotypes [18–21,54]. Furthermore, consistent with a recent meta-analysis investigating animal spatial personalities [54], we also found that the spatial behaviours of crocodiles were more repeatable than their social behaviour, indicating a greater degree of niche differentiation in how individuals use space compared to how they associate.

By tracking individual estuarine crocodiles across multiple consecutive years, we found that animals varied in how consistently they adopted a particular movement and social behaviour through time. Individual crocodiles varied in their degree of niche specialization, with some being behavioural specialists (low within-individual variation) adopting a narrow range of phenotypes, while others were more generalist (high within-individual variation) in the phenotypes they adopt. How specialized individuals are for a given phenotype may correlate with their behavioural niche [25,26]. Consistent with these studies, we also found that an individual’s degree of niche specialization correlated to their behavioural niche. Crocodiles, which on average were either more sociable or displayed greater site fidelity, were more specialized in their expression of these behaviours than those that were less sociable or had a lower site fidelity. However, it is unclear from our results what benefits (if any) individuals may gain by becoming either generalists or specialists in these behaviours.

One possibility is that by differing in their degree of behavioural specialization, individuals can mitigate competition and conflict with conspecifics, thereby increasing the stability of their social relationships and maximizing their fitness regardless of the phenotype they adopt [55,56]. By specializing in high sociability and high site fidelity phenotypes, individuals may also gain further fitness benefits from increased familiarity with conspecifics and their environment [57]. In African elephants (*Loxodonta africana*), increased habitat familiarity through time was found to increase the association rates between local and translocated individuals, with more social individuals displaying increased body condition [58]. Similarly, in roe deer (*Capreolus capreolus*) and black-tailed deer (*Odocoileus hemionus*), increased habitat familiarity was found to increase individual fitness through improved knowledge of resource distribution, refuge sites and predation risk within their environment [59,60]. Further research is therefore required to examine the influence of conspecific and habitat familiarity, along with potential physiological consequences (i.e. metabolic rate, stress), to determine the drivers of behavioural niche specialization in crocodiles.

An individual’s spatial and social behaviours are fundamental aspects of their ecology that are inherently linked [9]. Consistent with this, we found that the daily activity of crocodiles was negatively correlated to their sociability, with more active individuals being less sociable than those that adopt a more sedentary phenotype. For large-bodied predators such as crocodiles, conflict with conspecifics can be particularly costly, resulting in severe injuries or death [36]. Individuals, therefore, may be adopting more active phenotypes to actively avoid associating with conspecifics. Indeed, for many species, conspecific avoidance represents an important social strategy for minimizing/mitigating social conflict [5,11,61]. Consistent with this idea, we also found that while the number of overlapping conspecifics increased with home range size (and hence greater activity rates), the number of conspecifics that individuals associated with decreased as home range size increased suggesting that the observed behavioural syndrome represents an active conflict avoidance mechanism. Further research, however, is required to better investigate and disentangle the influence of conspecific avoidance on the spatial and social behaviours of estuarine crocodiles and other species.

Site fidelity and an increased familiarity between conspecifics are another potential conflict mitigation strategy reducing the costs associated with territorial defence and increasing individual fitness through the formation of stable communities [29,30,62,63]. For example, in red squirrels (*T. hudsonicus*), site fidelity increased an individual’s familiarity with neighbouring conspecifics resulting in the formation of stable communities [29,62]. By contrast, in estuarine crocodiles, we found no correlation between an individual’s degree of site fidelity and how sociable they were with conspecifics. This was surprising as previous work on estuarine crocodiles has suggested that site fidelity, and familiarity with conspecifics, plays a crucial role in mediating and maintaining the associations and spatial overlaps between individuals [37,38]. While no correlation was found between site fidelity and sociability, a behavioural syndrome was present between activity and site fidelity. Consistent with previous work investigating estuarine crocodile space use, site fidelity decreases as an individual’s mobility increased [32,34]. As a behavioural syndrome was also found between activity and sociability, our results suggest that rather than directly influencing sociability, that site fidelity in crocodiles may instead be a consequence of how individuals navigate their environment. Supporting this idea, we also found that site fidelity was correlated to body size, with larger crocodiles displaying a greater degree of site fidelity owing to their increased ability to defend space, while smaller crocodiles were excluded and thus must move more. However, further research using alternative measures of site fidelity (i.e. distance between home range centroids) and/or conspecific familiarity is required to gain further insights and disentangle the relationships between site fidelity, conspecific familiarity and sociability in crocodylians.

Overall, our results highlight that regardless of the degree of variation present either between or within individuals, the spatial and social behaviours of crocodiles are inherently linked at the individual scale. We found that not only do individual estuarine crocodiles differ in the spatial and social phenotypes they adopt but also in how consistently they adopt these phenotypes through time. We also found that how willing individuals are to associate with conspecifics was influenced by how active they are within their environment. Together, these findings demonstrate how accounting for both the inherent variation present between and within individuals can provide novel insights into the spatial–social interface that are not possible when solely focusing on the average differences present between individuals and species.

## Data Availability

The raw and processed data, along with all of the R code supporting the analyses reported in this article are available at [64]. Supplementary material is available online [65].
